# Canine vector-borne parasites in the Galapagos

**DOI:** 10.1186/s13071-024-06592-z

**Published:** 2024-12-18

**Authors:** Carla Andreea Culda, Nathalie G. Tejena Rodriguez, Fortunato Puleo, Rafael M. Polit Sosa, Luciana Cătălina Panait, Cristina Daniela Cazan, Georgiana Deak, Renato Leon, Rommel Lenin Vinueza, Diego Páez-Rosas, Andrei Daniel Mihalca

**Affiliations:** 1 Department of Parasitology and Parasitic Diseases, University of Agricultural Sciences and Veterinary Medicine of Cluj-Napoca, Cluj-Napoca, Romania; 2 Universidad Regional Amazonica Ikiam, Tena, Ecuador; 3 Faculty of Veterinary Medicine, University of Life Sciences “King Mihai I” of Timișoara, Timișoara, Romania; 4 Escuela de Medicina Veterinaria, Cumbayá, Universidad San Francisco de Quito, Quito, Ecuador; 5 Department of Public Health and Food Hygiene, University of Agricultural Sciences and Veterinary Medicine of Cluj-Napoca, Cluj-Napoca, Romania; 6 Laboratorio de Entomología Médica & Medicina Tropical LEMMT, Universidad San Francisco de Quito, Quito, Ecuador; 7 Universidad San Francisco de Quito, Galapagos Science Center, Puerto Baquerizo Moreno, Isla San Cristóbal Ecuador; 8 Dirección del Parque Nacional Galápagos, Unidad Técnica Operativa San Cristóbal, Galápagos, Isla San Cristóbal Ecuador

**Keywords:** Domestic dogs, Heartworm, Molecular biology, Invasive species, Endemic species

## Abstract

**Background:**

The extraordinary Galapagos Islands, with an impressive number of endemic and native species, maintain the interest and curiosity for researchers from all over the world. The native species are known to be vulnerable to new pathogens, cointroduced with their invasive hosts. In the case of invasive parasitic arthropods, their evolutionary success is related to the association with other invasive hosts (such as domestic animals). These associations could become a significant driver of change, as occasionally they can seek another hosts and have the capacity to transmit pathogens between domestic and wild animals. The current study aims to identify the distribution and abundance of canine vector-borne parasites in the Galapagos Islands based on the possibility that some of them could spill over to endemic mammals.

**Methods:**

A total of 1221 blood samples were randomly collected from privately owned dogs on San Cristóbal, Isabela, Santa Cruz, and Floreana Islands during the years 2021 and 2022. All samples were examined for vector-borne pathogens using the modified Knott’s test and conventional, nested, and multiplex polymerase chain reactions (PCRs), followed by sequencing.

**Results:**

The PCR and Knott tests confirmed the presence of *Dirofilaria immitis* (2%, 25/1221) in all islands. While molecular analyses showed heartworm DNA only in dogs from the San Cristóbal (3.3%) and Isabela (2.4%) Islands. Moreover, other pathogens *Babesia vogeli* (3%, 37/1221) and *Hepatozoon canis* (0.2%, 2/1221) were detected for the first time by molecular analyses.

**Conclusions:**

Dogs from Galapagos are hosts to various pathogens, of which some are potentially zoonotic while some other could be spill-over to endemic endangered carnivores, such as sea lions. To understand and limit their impact, long-term surveillance, control, and awareness is needed.

**Graphical abstract:**

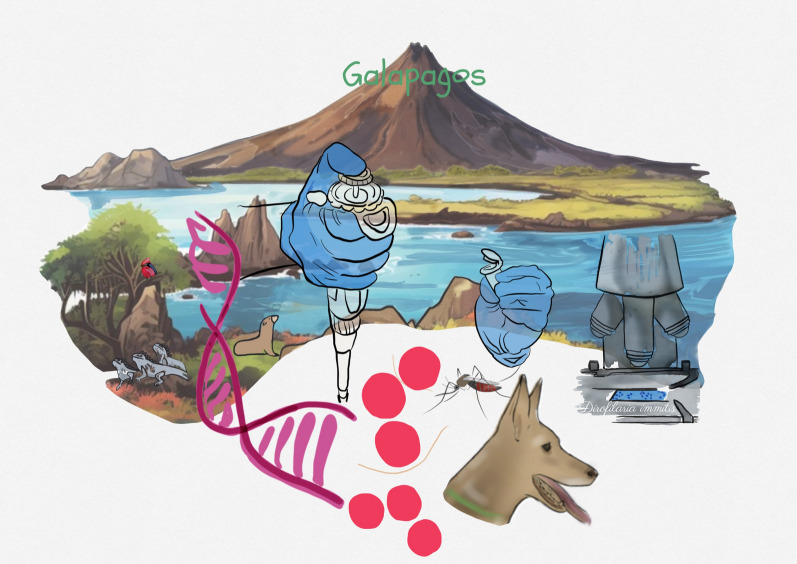

**Supplementary Information:**

The online version contains supplementary material available at 10.1186/s13071-024-06592-z.

## Background

The Galapagos Islands have an impressive number of endemic species and maintained the interest of researchers from all over the world, being also named by UNESCO “a living museum and showcase of evolution.” Therefore, this archipelago is one of the world’s most strictly protected natural areas, as it has many threatened and endangered species [1, 2]. Despite their remoteness, invasive species have been introduced intentionally or unintentionally to the islands throughout the time. Native species are known to be vulnerable to new pathogens cointroduced with their invasive hosts [2, 3]. For instance, invasive insects constitute 23% of the total insect species [4] in the archipelago.

In the case of invasive parasitic arthropods, their evolutionary success is related not only to abiotic factors but also to the association with other invasive hosts, such as domestic animals [2, 5]. These associations could become a significant driver of change, as occasionally they can switch hosts and have the capacity to transmit pathogens between domestic and wild animals [6]. It is hard to determine when the first invasive parasites were introduced to Galapagos [2]. However, over time, vector-borne disease transmission by introduced or indigenous arthropods in which domestic dogs (i.e., could be hosts to various pathogens) can have negative consequences for various native and endemic species, disrupting the cycle of entire ecosystems [2, 6–8].

Eleven species of hard ticks are present in the Galapagos, three which are introduced and eight are endemic [9–11]. In the case of fleas, there are no data on their diversity, distribution, or hosts. The only flea in Galapagos, *Parapsyllus cedei*, was recorded in nests of seabirds in Genovesa and Santa Cruz and is considered to be native [12–14]. Mosquitoes (Culicidae) represent another important group of vectors in this fragile ecosystem. Three species of mosquitoes are known in Galapagos: two of them (*Aedes aegypti* and *Culex quinquefasciatus*) are introduced and one (*Aedes taeniorhynchus*) is considered to be native [15–19].

Marine endemic mammals of Galapagos, the Galapagos fur seal (*Arctocephalus galapagoensis*) and Galapagos sea lion (*Zalophus wollebaeki*), were isolated from many of the mainland carnivore-associated pathogens due to their remote geographic position [20]. Generally, due to coevolution, native parasites and microbes are not pathogenic to their hosts; however, for introduced pathogens, native species lack an immune response [2, 21]. Few studies are available on the impact of multihost pathogens on endemic fauna, although the same threats can affect native species and humans in different ways. The current study aims to identify the distribution and abundance of canine vector-borne parasites in the Galapagos Islands based on the possibility that some of them could spill over to endemic mammals. Genetic characterization aimed to identify the pathogens that are present in the Galapagos Islands and could have an impact on resident fauna.

## Methods

### Sample collection

**Fig. 1:**
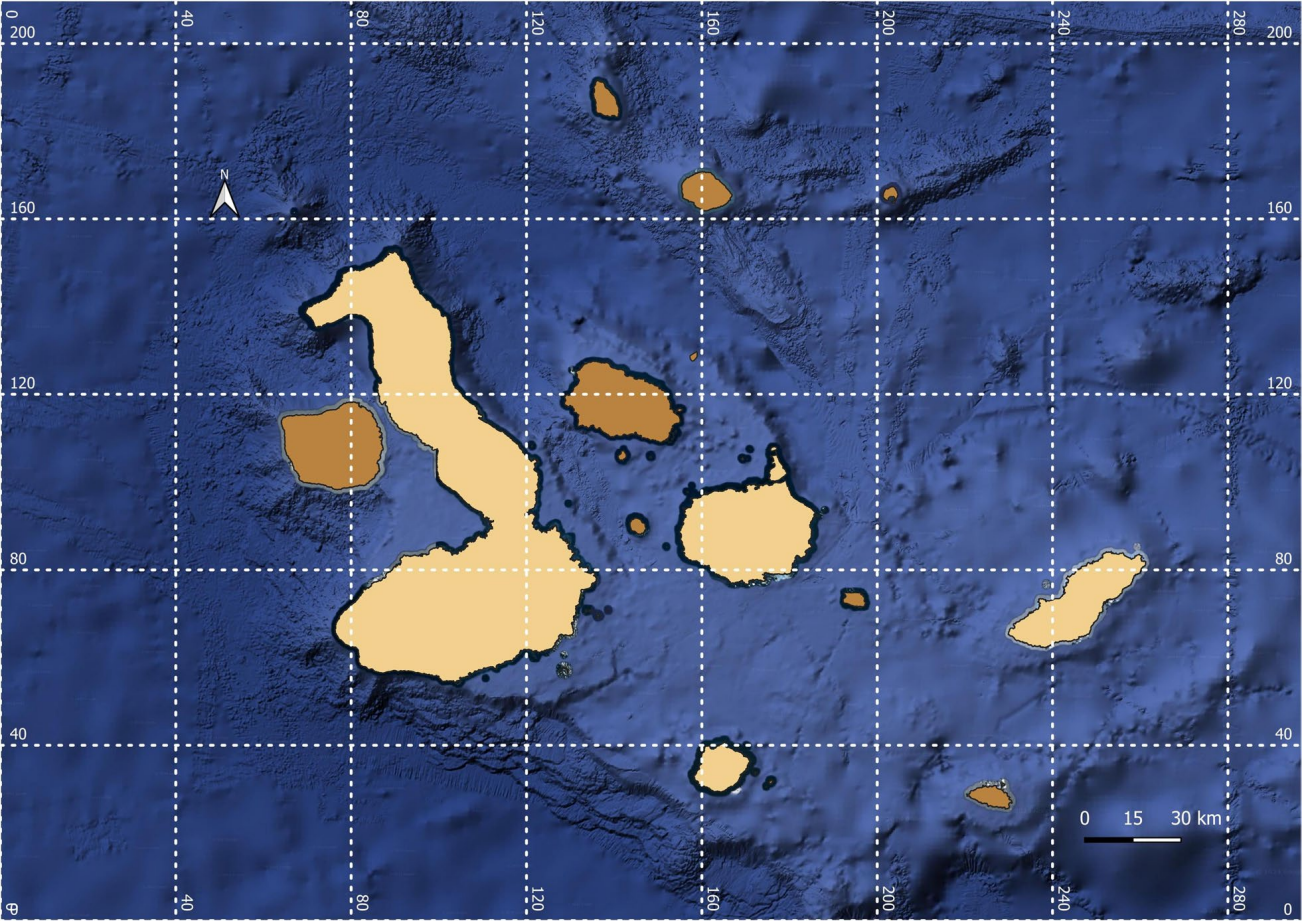
Islands from where samples were collected (San Cristóbal, Isabela, Santa Cruz, and Floreana), in light brown

The sampling was conducted over a period of 2 years, between July and September 2021 (San Cristóbal Island) and between July and August 2022 (Isabela, Santa Cruz, and Floreana Islands), corresponding in both cases to the dry season. A total of 1221 dogs (652 males and 569 females) from four human-inhabited islands were examined for vector-borne parasites (Fig. 1 and Table 1).

**Table 1:** Distribution of sampled dogs according to the location and category

Category	Isabela	Floreana	Santa Cruz	San Cristóbal	Total
Owned dogs	168	22	341	603	1134
Animal shelters	0	0	16	0	16
Veterinary clinics	44	0	27	0	71
Urban	158	17	315	547	1037
Rural	54	5	69	56	184
Total	212	22	384	603	1221

The blood samples were collected from the cephalic vein of the foreleg or the saphenous vein of each dog using S-Monovette an EDTA K2E system (SARSTEDT AG and Co., Germany). All blood samples were stored at 4 °C until further analysis but for no longer than 12 h. In parallel, 0.5 mL of whole blood was taken, mixed with 96% ethanol, and was stored at −20 °C until further molecular processing.

### Blood examination

All blood samples collected in Isabela, Santa Cruz, and Floreana Islands were analyzed for the presence of microfilariae in circulating blood using the modified Knott’s test [22–26], while the results of the Knott’s test from San Cristóbal Island are already published [26].

### DNA extraction and PCR amplification

The DNA was extracted from the ethanol preserved blood using the DNeasy Blood and Tissue Kit (Qiagen, Germany), according to the manufacturer’s instructions. The DNA concentration and purity (260/280 ratio) were assessed in duplicates using a NanoDrop ND-1000 spectrophotometer (NanoDrop Technologies, Inc., Wilmington, DE) on 5% of randomly selected samples.

Conventional polymerase chain reaction (PCR) protocols were used to assess genomic DNA of vector-borne pathogens in blood samples using the techniques described in Supplementary File 1. The DNA extracted from blood samples was processed by multiplex PCR (mPCR) targeting several filaroid species that infect dogs (*D. immitis*, 170 bp; *D. repens*, 480 bp; and *A. reconditum*, 590 bp; [27]). For improved specificity, the positive samples obtained from the mPCR assay were further tested using NTF/NTR primers combination, following the techniques outlined in [28]. This molecular test amplifies a partial sequence of the cytochrome c oxidase subunit 1 (*cox1*) gene of spirurid nematodes, generating products with longer sequences. To assess the possible presence of other filariae that infect dogs, a partial fragment of the ITS2 region was amplified. The amplification procedure was conducted using conventional PCR with pan-filarial primers (DIDR-F1/DIDR-R1) described by ref. [29].

To detect blood Apicomplexan hemoparasites (*Babesia* spp., *Theileria* spp., and *Hepatozoon* spp.), a nested PCR protocol was used for initial screening with the primers BTH-1F and BTH-1R and GF2 and GR2 [30–32]. All positive or doubtful samples were subsequently screened by specific nested PCR assays targeting the 18S rDNA of *Babesia* spp. [33] and the mitochondrial cytochrome b (*cytb*) and *cox1* genes of *Babesia* spp. [34] and *Hepatozoon* spp. [35] (Supplementary File 1).

For each reaction, a positive control with DNA and a negative control without DNA were added to evaluate the reaction’s specificity and determine the presence of any contaminants. Positive control consisted of DNA that had been confirmed to be positive for the targeted pathogens. Thus, the positive controls for *Dirofilaria* spp. genes are available in GenBank under the following accession numbers: KT716014 for *D. immitis*, and MW656250, and MW656251 for *Dirofilaria repens*. Furthermore, for *Babesia* spp., the positive control accession numbers are MW939359 (18S rDNA), MW938761 (*cytb* gene), and MW938763 (*cox1* gene). The positive control for *Hepatozoon* spp. (18S) was derived from *Hepatozoon* sp. obtained from a spleen sample of a wild cat in Romania. Although this sequence has not been submitted to GenBank, it is 100% similar to accession number OM256568, with 99% query coverage. PCR products were visualized on 1.5% agarose gels using ECO Safe Nucleic Acid Staining Solution (PacificImage Electronics, New Taipei City, Taiwan).

The amplicons obtained were purified with the Gel/PCR DNA Fragments Extraction Kit (Geneaid Biotech Ltd., New Taipei City, Taiwan) according to the manufacturer’s instructions and sequenced bidirectionally (Macrogen Europe, Amsterdam, the Netherlands). Subsequently, all sequences were subjected to analyses and edited using Geneious^®^ 4.85 software [36]. The sequences underwent comparison with those present in GenBank™ using the local Basic Local Alignment Search Tool (BLASTn) analyses.

### Definition of positive results

For the epidemiological statistical analysis, the final status for the *D. immitis* infection was determined based on the outcomes of microscopic (Knott’s test) and molecular testing performed for common filarial nematodes. The molecular processing involved both mPCR and conventional PCR to obtain a comprehensive profile of the common filarial nematodes (panfilarial—nine species could be detected). We retested positive amplicons from the mPCR for sequencing using the mitochondrial *cox1* gene. Therefore, the Knott’s test defined the positive results for *D. immitis* as the presence of microfilaria in the circulating blood under microscopic examination, even if the PCR results were negative. Similarly, samples that tested positive in one, two, or all three molecular tests without microscopic detection of microfilaria were considered positive results.

The positive results for *Babesia vogeli* were determined by either two positive molecular tests out of the three species-specific tests or by one test with a positive result that was confirmed by sequencing. Regarding the positive *Hepatozoon canis* result, it was concluded by the use of a specific molecular target gene, followed by sequencing. The positive molecular results indicate dogs that tested positive for the target pathogens.

### Statistical analysis

Statistical analysis was performed using RStudio 2023.09.1. The prevalence values were compared through the Fisher’s exact test on the islands where positive animals were found and the free roam condition on each of these islands. A generalized linear model (GLM) from the stats “R package” was applied to determine if the variables: housing, island, location, sex, and free roam are correlated with the positivity in dogs. Odds ratios (ORs), 95% confidence intervals (CI), and *P* values were calculated for epidemiological statistical analysis. When the value of OR is equal to 1, it indicates no association between the exposure and the outcome. Statistical significance was established by a *P* value of less than 5% (0.05) and a confidence level of 95%.

The prevalence values were compared through the Fisher’s test on the islands where positive animals were found and the free roam condition on each of these islands. The predictive values of the tests used (Knott test and *cox1* gene filarial nematodes) were calculated using mPCR as the gold standard. To evaluate the usefulness and applicability of these tests, we calculated the sensitivity and specificity [37]. Finally, a probability model was developed using the PROC package and the receiver operating characteristic (ROC) curve was plotted, with the area under the curve (AOC) calculated.

A generalized linear model (GLM) from the “Stats” package was applied to determine if the variables such as housing, island, location, sex, and free roam are correlated with the positivity to *D. immitis* and *B. vogeli* in dogs.

## Results

### Roaming behavior

In our study, 25.2% of dogs with owners roam freely. The Floreana, Isabela, and San Cristóbal Islands were the ones that mostly showed this behavior, 40% (9/22), 30.6% (65/212), and 29.7% (179/603), respectively, while on Santa Cruz Island only 14.3% (35/384) of owned dogs roamed freely. Although Santa Cruz Island has the largest human and dog population, the tendency of dogs to roam unrestricted was statistically lower than on San Cristobal Island (*P* = 0.001, OR 0.001) and Isabela Island (*P* = 0.001, OR 5.29), where positive cases were documented.

### *D. immitis*

Overall, 25 out of 1221 blood samples (2.0%) were positive for *D. immitis* (Table 2). The DNA sequences from the 25 positive samples revealed similarity (97–100%) with the available DNA sequences of *D. immitis* at NCBI GenBank (Supplementary File 2). The high-quality sequences obtained were submitted to the GenBank international database under the accession number (PQ044876-PQ044877, *Q* > 20 value higher then 90; Supplementary File 3).

**Table 2:** Comparative analysis of *D. immitis* prevalence in dogs

Variable	Category	No. of samples	Positive (%)	*P* value	OR	95% CI
Total		1221	25 (2.0)			1.3–2.9
Sex	Males	652	16 (2.5)	0.3837	1.5653 NA	1.3–3.8
	Females	569	9 (1.6)			0.6–2.6
Age	<1 Year	149	2 (1.4)	0.1441	NA	0–3.2
	1–4 Years	648	9 (1.4)			0.5–2.3
	4–10 Years	345	12 (3.5)			1.6–5.4
	>10 Years	50	2 (4)			0–9.4
	Unknown	29	0			0
Breed	Pure breed	385	1 (0.3)	0.0055^*^	0.0881	0–0.8
	Mixed breed	836	24 (2.9)			1.7–4
Environment	Urban	1013	21 (2.1)	1	1.0796 NA	1.2–3.0
	Rural	208	4 (1.9)			0.1–3.8
Housing	Outdoor	560	18 (3.2)	0.0284^*^	NA	1.8–4.7
	Indoor	231	3 (1.3)			0–2.8
	Outdoor and indoor	430	4 (1)			0.1–1.9
Free roaming	Yes	308	7 (2.3)	0.9282	1.1563 NA	0.6–4
	No	913	18 (2)			1.1–2.9

*Statistically significant, *OR* odds ratio, *95% CI* 95% confidence interval, *NA* not applicable

**Fig. 2:**
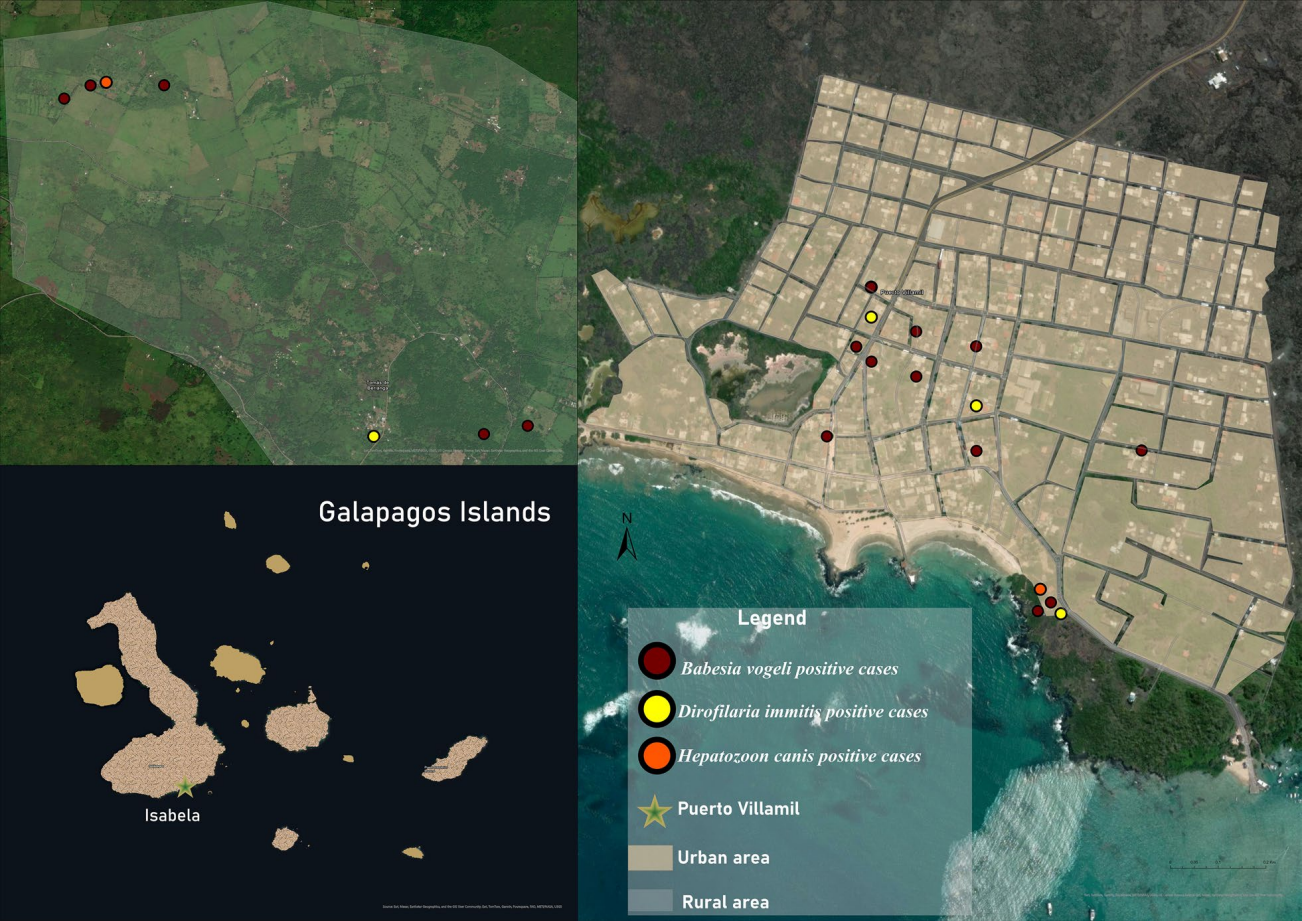
The spatial distribution of positive dogs on Isabela Island

**Fig. 3:**
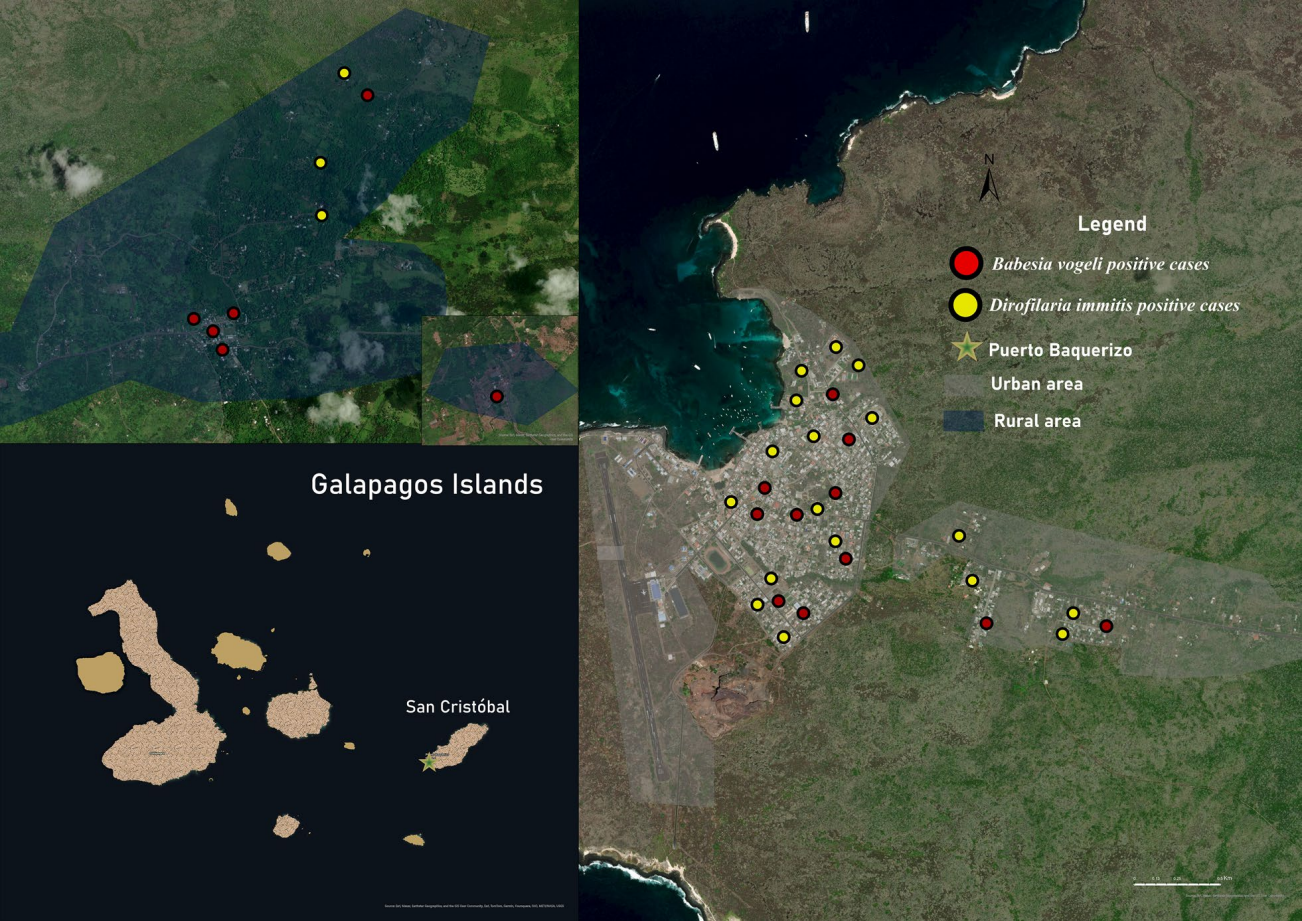
The spatial distribution of positive dogs on San Cristóbal Island

The prevalence of heartworm in domestic dogs by island was as follows: Isabela 2.4%, Floreana 0%, Santa Cruz 0%, and San Cristóbal 3.3% (Figs. 2 and 3). The sample analysis revealed that 19 of the examined dogs tested positive (1.6%) using mPCR. Out of these, 14 dogs also showed positive results (1.2%) when their *cox1* gene sequences were analyzed. These sequences were found to be 97–100% similar to different sequences of *D. immitis* in the GenBank sequence database. Furthermore, we confirmed the presence of this species by amplifying and analyzing the 5.8S-ITS2-28S rDNA gene fragment.

The *cox1* gene filarial nematodes test showed greater sensitivity than the Knott’s test, while the Knott’s test showed greater specificity than the *cox1* gene filarial nematodes test (Table 3).

**Table 3:** Sensitivity and specificity of the Knott versus molecular diagnosis of *D. immitis* using mPCR as the gold standard

Method	TP	TN	FN	FP	Se (%)	Sp (%)	AUC
Knott	8	1160	10	6	57	99.1	99.7
*cox1*NTF/NTR	14	4	7	2	87.5	36.3	69.5

*TP* true positives, *TN* true negatives, *FN* false negatives, *FP* false positives, *Se* sensitivity, *Sp* specificity, *AUC* *Area under the curve*

However, when applying a GLM, no significant relationship was found between variables examined and positivity. The influence of the variables housing, island, and sex could not be related to the presence of *D. immitis* (Supplementary File 4).

### Apicomplexan hemoparasites

**Fig. 4:**
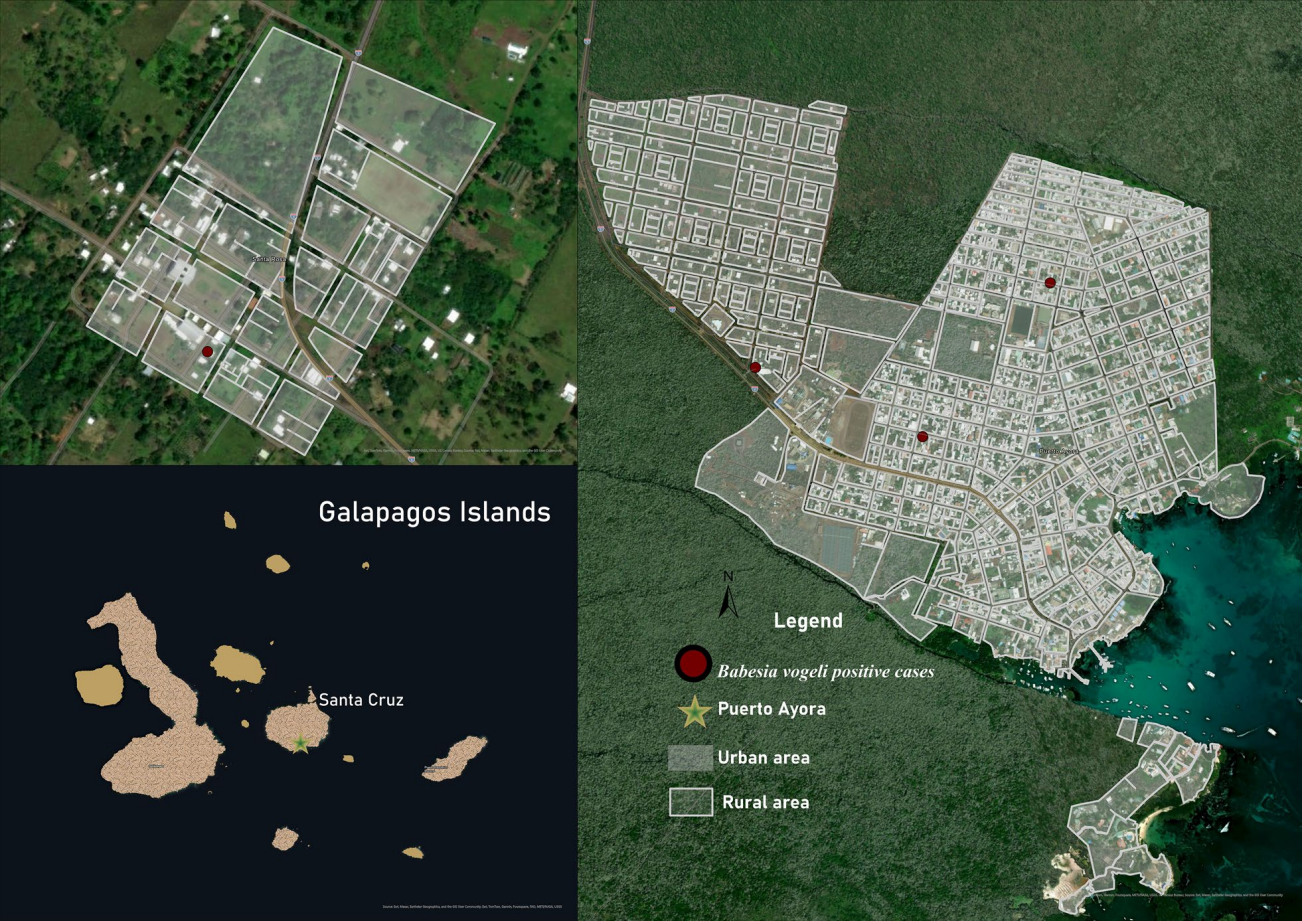
The spatial distribution of positive dogs on Santa Cruz Island

The BLAST analysis of the 18S rDNA sequences showed a 97–100% identity with different sequences of *Babesia* spp., *Hepatozoon* spp., and *Theileria* spp. in 67 dogs (5.5%, *n* = 1221). To further validate the positive results, species-specific nPCR assays were performed targeting the 18S rDNA of *Babesia* spp. (Supplementary File 5). The sequence analysis showed that 37 dogs tested positive for *B. vogeli* according to the sequences (95–100%) (Table 4 and Figs. 2, 3 and 4). The high-quality sequences obtained are available online under the accession numbers PQ136446–PQ136456; *Q* > 20 higher then 95%.

**Table 4:** Comparative analysis of *B. vogeli* prevalence in dogs

Variable	Category	No. of samples	Positive (%)	*P* value	OR	95% CI
Total		1221	37 (3)			2.1–4
Sex	Males	652	19 (2.9)	0.9313	0.9188	1.7–4.2
	Females	569	18 (3.2)			1.8–4.6
Age	<1 Year	149	12 (8.1)	0.0002^*^	NA	3.7–12.4
	1–4 Years	648	21 (3.3)			1.9–4.6
	4–10 Years	345	1 (0.3)			0–0.9
	>10 Years	50	2 (4)			0–9.4
	Unknown	29	1 (3.5)			0–10.1
Breed	Pure breed	385	9 (2.4)	0.4363	0.6907	1–3.9
	Mixed breed	836	28 (3.4)			2.1–4.6
Environment	Urban	1013	26 (2.6)	0.0624	0.4718	1.6–3.5
	Rural	208	11 (5.3)			2.3–8.3
Housing	Outdoor	560	27 (4.8)	0.0034^*^	NA	3.1–6.6
	Indoor	231	4 (1.7)			0.1–3.4
	Outdoor and indoor	430	6 (1.4)			0.2–2.5
Free roaming	Yes	308	11 (3.6)	0.6538	1.2635 NA	1.5–5.6
	No	913	26 (2.8)			1.8–3.9

*Statistically significant, *OR* odds ratio, *95% CI* 95% confidence interval, *NA* not applicable

However, further confirmation revealed that only 28 samples showed the presence of this species via *cytb* gene amplification and analysis and only 24 samples through *cox1* gene amplification and analyses. The sequences obtained from domestic dogs matched 99–100% with *B. vogeli* sequences from GenBank (available in Supplementary File 5). The sequences obtained from the *cytb* and *cox1* gene are available in Supplementary File 3.

The GLM analysis revealed that none of the variables examined were significantly associated with the presence *B. vogeli* (Supplementary File 4).

Two samples from Isabela Island tested positive for *H. canis* (0.2%) (Fig. 2). One case was from the rural area, and the other was from the urban area. Both sequences matched 99.70% with the *H. canis* sequences stored in GenBank (MN393911, Cuba). All sequences obtained from the positive samples were deposited in GenBank international database under the accession numbers: PQ136654–55 and PQ136656–57; *Q* > 20 higher than 95%.

## Discussion

Our work extends completes the data of the previous studies about the pathogens introduced in the Galapagos archipelago (Table 5). As reviewed by [38], most of the invasive parasites tend to infect and to be more virulent in native hosts, due to the lack of natural resistance.

**Table 5:** Literature review of vector-borne pathogens reported in dogs in Galapagos

Island	Pathogen	Prevalence (%)	Method	References
San Cristóbal	*D. immitis*	1.7	Knott test	[26]
		3.3	PCR	Present study
	*Babesia* *vogeli*	2.8	PCR	Present study
	*H. canis*	–	PCR	Present study
Floreana	*D. immitis*	77	Antibody	[39]
		87	Knott test	[39]
		14.8	Necropsy	[39]
		–	Knott test	Present study
		–	PCR	Present study
	*Babesia* *vogeli*	–	PCR	Present study
	*H. canis*	–	PCR	Present study
Santa Cruz	*D. immitis*	–	Antigen	[40]
		–	Antigen	[41]
		6.9	Antigen	[11]
		Not given	PCR	[11]
		Not given	Diff-Quik	[11]
		–	Knott test	Present study
		–	PCR	Present study
	*Babesia* *vogeli*	1.05	PCR	Present study
	*H. canis*	–	PCR	Present study
Isabela	*D. immitis*	Not given	Necropsy^1^	[42]
		34	Antigen	[43]
		1.9	Knott test	Present study
		2.4	PCR	Present study
	*Babesia *spp.	–	Antibody	[43]
	*Babesia* *vogeli*	7.6	PCR	Present study
	*H. canis*	1.0	PCR	Present study

^1^Feral dogs

The canine heartworm, *D. immitis* is particularly important, as it can cause disease in dogs and other wild carnivores and also infect humans [44, 45]. The heartworm was reported in the Galapagos Islands for the first time in the 1980s [39, 42] and occasionally also later (Table 5). Furthermore, a recent study [8] reported the presence of 20 heartworms in a male sea lion on Santa Cruz Island. On Floreana Island, local people tested positive for antibodies, and it was shown that the sea lions that inhabit this island had circulating microfilariae [39]. The different results obtained on Floreana Island reflect changes over time. Several factors could explain the absence of *D. immitis*, such as lower mosquito densities, lower movement of dogs from other islands, and mainly the low population size of dogs. Additionally, they tested 25 young sea lions on San Cristóbal Island for *D. immitis* antigen, where 8% tested positive [8].

Concerning the risk factors, the prevalence of *D. immitis* showed that males had a slightly higher prevalence (2.5%) compared with females (1.6%), although this difference was not statistically significant (*P* = 0.3837), suggesting that sex may not be a determinant for the presence of the parasite. In contrast, age significantly influenced positivity, with dogs aged 4–10 years showing the highest prevalence (3.5%), possibly due to the longer time of exposure.

However, the GLM results did not establish whether there was a significant interaction between these variables in relation to positivity. A study conducted in Bucaramanga, Colombia, found a prevalence of 10.82% of *D. immitis* in dogs [46], highlighting the importance of demographic and environmental factors in the spread of the disease [46–48].

Molecular methods have a higher sensitivity to detect microfilariemia in canine blood samples, even at low levels [49–54]. Therefore, the difference between the results obtained via the Knott test and molecular test could be due to low microfilariemia. An important aspect of the results from combining microscopic and molecular methods is that together they can improve the accuracy of the diagnosis [53]. The molecular methods employed in this study were chosen to enhance results sensitivity, as well as the characterization and differentiation of filarial species. Two molecular tools targeting the *cox1* gene (mPCR and conventional PCR) were utilized concurrently to enhance the quality of the obtained positive sequences. The third method was employed to detect the presence of other filarial species (*A. dracunculoides*; *B. pahangi*; *B. malayi*; *B. timori*; *M. ozzardi*; *O. volvulus*) in the extracted DNA, targeting the ITS2 gene [54].

The presence of vectors from the Culicidae family favors a complete cycle of *D. immitis* [26, 55–57]. Given that canine heartworm is a mosquito-borne filarial nematode and three species of mosquitoes capable of transmitting the parasite are found on these islands [11, 15, 16, 18, 19, 39, 58], we should consider the risk posed to pinnipeds, which often live close to dogs and mosquitoes.

Human settlements on the San Cristóbal, Santa Cruz, and Isabela Islands present a great risk of disease transmission from domestic carnivores to Galapagos sea lions [8, 59–61]. On each island, domestic dogs are present and they are often allowed to roam the streets and beaches [62]. This behavior can increase the risk of infection transmission, added to the fact that dogs probably do not receive regular antiparasitic treatments (Mihalca and Culda, personal observation).

Another essential aspect of this study was to provide the first demonstration of *B. vogeli* and *H. canis* in dogs in Galapagos. Ticks represent the second disease vector group after mosquitoes. They are frequently considered as a growing threat to human and animal health on worldwide [63]. It is known that they have the ability to transmit Apicomplexa (*Babesia*, *Theileria*, and *Hepatozoon*), which can cause pathogenicity in the affected host [64, 65]. Previous studies showed that ticks collected from dogs in Galapagos were *Rhipicephalus sanguineus* sensu lato [11, 40, 41, 43]. However, all these studies were done before the recent taxonomic agreement [66], which concluded that the so called “tropical linage” of *R. sanguineus* s.l. should be regarded as *R. linnaei*.

The 18S rDNA gene is a highly conserved region that plays an essential role in the molecular diagnosis of piroplasmids [67–75]. Nevertheless, several studies suggested that detecting multiple pathogens in a single sample may have some limitations and variables in the target size gene’s amplification efficiency [74, 76, 77].

*B. vogeli* is an emerging pathogen of dogs, with a worldwide distribution, particularly in tropical and subtropical countries [72, 78–80]. In our case, the highest prevalence was obtained in dogs younger than 1 year old (8.1%; 12/1,221) and in those aged 1–4 years (3.3%; 21/1,221). This may be related to higher exposure to ticks and mainly to the lack of immunity, as known for *Babesia* spp. in animals following multiple exposure [80, 81]. Several studies evaluated the prevalence of this pathogen in dogs across different continents, and its presence seems to be more common in warm climate areas. Australia, Cambodia, Thailand, Egypt, and Costa Rica have the highest prevalence of *B. vogeli* [72, 80]. Moreover, it is interesting that *B. vogeli* was detected also in wild carnivores in other regions, such as Brazil [79, 82], Thailand [80, 83], Zimbabwe [80, 84], South Africa [80, 84], and Tanzania [80, 85].

Regarding *B. vogeli* risk factors, the study revealed a slightly lower prevalence in males (2.9%) compared with females (3.2%), although this difference was not statistically significant (*P* = 0.9313), suggesting that sex is not a determinant factor for the presence of the parasite. Conversely, age had a significant influence on positivity, with dogs under 1 year of age showing the highest prevalence (8.1%) (*P* = 0.0002), possibly due to a higher susceptibility to infection.

Similar to the case of *D. immitis*, differences in prevalence were found according to breed, with a lower prevalence in pure breeds (2.4%) compared with mixed breeds (3.4%), although this difference was not statistically significant (*P* = 0.4363). The higher prevalence observed in dogs living outdoors (4.8%) compared with those living indoors (1.7%) or having access to both indoor and outdoor environments (1.4%) (*P* = 0.0034) is possibly due to greater exposure to ticks. A study by Zygner et al. [80] highlighted that the most important risk factors for *Babesia* infection include living in rural areas, in kennels or animal shelters, or in regions endemic for the infection, as well as tick infestation and lack of acaricide treatment. The characteristics of populated areas in the Galapagos Islands resemble a rural environment, which would be related to what was mentioned by this author. Regarding the GLM, as in the case of *D. immitis*, the results did not allow the establishment of any significant interaction between these variables in relation to positivity, likely requiring a larger sample size or the investigation of other variables.

Finally, it was also possible to identify *H. canis*, another tick-borne parasite, which has been recognized in South America as a significant threat to the well-being of dogs, especially in rural areas of Brazil and Costa Rica [86–90].

## Conclusions

Our study demonstrated the presence, although with low prevalence, of canine vector-borne parasites in Galapagos. Among the tested pathogens, *D. immitis* is particularly relevant from a conservation medicine perspective, as it can be transmitted and be pathogenic to the endangered Galapagos sea lion. Our study also brings new data on the global distribution of *B. vogeli* and *H. canis*, demonstrating for the first time their presence in Galapagos.

## Supplementary Information

Supplementary Material 1. Primers sets used for the amplification of DNA.

Supplementary Material 2. BLAST comparisons between the obtained sequences and the GenBank sequences.

Supplementary Material 3. Consensus sequences derived from the obtained sequences.

Supplementary Material 4. GLM analysis results for Dirofilaria immitis and Babesia vogeli in dogs.

Supplementary Material 5. BLAST comparisons between the obtained sequences and the GenBank sequences.

## Data Availability

Data are provided within the manuscript and in the Supplementary Information files. Our accesion numbers include: PQ044876-PQ044877 for *D. immitis*, PQ136446–PQ136456 for *B. vogeli*, PQ136654–PQ136655 and PQ136656–PQ136657 for *H. canis*.

## Abbreviations

DNA: Deoxyribonucleic acid
PCR: Polymerase chain reaction
mPCR: Multiplex PCR
nPCR: Nested PCR
rDNA: Ribosomal RNA
NCBI: National Center for Biotechnology Information
BLAST: Basic Local Alignment Search Tool
