# Supplementary material for: Canine vector-borne parasites in the Galapagos
Source: Parasit Vectors. 2024 Dec 18;17:515. doi: 10.1186/s13071-024-06592-z (PMC11656949; doi:10.1186/s13071-024-06592-z)
Supplement: Supplementary file 1 — Supplementary Material 1. Primers sets used for the amplification of DNA. [file 13071_2024_6592_MOESM1_ESM.docx]

Primers sets used for the amplification of DNA

| **Primer** | **Sequence (5′-3′)** | **Target gene** | **Amplicon size (bp)** | **Annealing temperature** | **Reference** |
| --- | --- | --- | --- | --- | --- |
| DiCox | ACCGGTGTTTGGGATTGTTA | *cox1* gene of filarial nematodes  (including *D. immitis*; *D. repens*; *A. reconditum*) | *D. immitis*, 170  *D. repens*, 480  *A. reconditum*, 590 | 52°C | [27] |
| DrCox | GTATAATTTTGGGTTTACATACTGTA |  |  |  |  |
| ArCox | ATCTTTGTTTATGGTGTATC |  |  |  |  |
| NTR | ATAAGTACGAGTATCAATATC |  |  |  |  |
| NTF | TGATTGGTGGTTTTGGTAA | mitochondrial *cox1* gene filarial nematodes | Filarioid nematodes, 670 | 47°C | [28] |
| NTR | ATAAGTACGAGTATCAATATC |  |  |  |  |
| DIDR-F1 | AGTGCGAATTGCAGACGCATTGAG | 5.8S-ITS2-28S  (including *D. immitis*; *A. reconditum*; *D. repens*; *A. dracunculoides*; *B. pahangi*; *B. malayi*; *B. timori*; *M. ozzardi*; *O. volvulus*) | *D. immitis*, 542  *A. reconditum*, 578  *D. repens*, 484  *A. dracunculoides*, 584  *B. pahangi*, 664  *B. malayi*, 615  *B. timori*, 625  *M. ozzardi*, 430  *O. volvulus*, 470 | 63°C | [29] |
| DIDR-R1 | AGCGGGTAATCACGACTGAGTTGA |  |  |  |  |
| BTH-1F | CCTGAGAAACGGCTACCACATCT | 18S ribosomal RNA of Piroplasmids | Apicomplexa, *Babesia* spp., *Theileria* spp., 561-613 | 60°C | [30 - 32] |
| BTH-1R | TTGCGACCATACTCCCCCCA |  |  |  |  |
| **Nested PCR:** |  |  |  |  |  |
| GF2 | GTCTTGTAATTGGAATGATGG |  |  | 50°C |  |
| GR2 | CCAAAGACTTTGATTTCTCTC |  |  |  |  |
| Bc_F1 | CGTAGTTGTATTTTTGCGT | 18S ribosomal RNA of *Babesia* spp. | *Babesia* spp., 370-380 | 50°C | [33] |
| GR2 | CCAAAGACTTTGATTTCTCTC |  |  |  |  |
| **Nested-PCR:** |  |  |  |  |  |
| Bc_F2 | CATTTGGTTGGTTATTTCGTTTT |  |  | 53°C |  |
| Bc_R1 | GTTCCTGAAGGGGTCAAAAA |  |  |  |  |
| Bc_cytb_F1 | TGGTCWTGGTATTCWGGAATG | *cytb* gene of *Babesia* spp. | *Babesia* spp., 580 | 50°C | [34] |
| Bc_cytb_R1 | AAGMYARTCTYCCTAAACATCC |  |  |  |  |
| **Nested PCR:** |  |  |  |  |  |
| Bc_cytb_F2 | RATKAGYTAYTGGGGAGC |  |  | 48°C |  |
| Bc_cytb_R2 | GCTGGWATCATWGGTATAC |  |  |  |  |
| Bab_For1 | ATWGGATTYTATATGAGTAT | *cox1* gene of *Babesia* spp. | *Babesia* spp., 980 | 45°C | [34] |
| Bab_Rev1 | ATAATCWGGWATYCTCCTTGG |  |  |  |  |
| **Nested PCR:** |  |  |  |  |  |
| Bab_For2 | TCTCTWCATGGWTTAATTATGATAT |  |  | 49°C |  |
| Bab_Rev2 | TAGCTCCAATTGAHARWACAAAGTG |  |  |  |  |
| HAM1F | GCCAGTAGTCATATGCTTGTC | 18S ribosomal RNA of *Hepatozoon* spp. | *Hepatozoon* spp., 1600 | 52°C | [35] |
| HPF2R | GACTTCTCCTTCGTCTAAG |  |  |  |  |
| **Nested PCR:** |  |  |  |  |  |
| EF-M | AAAACTGCAAATGGCTCATT |  |  | 55°C |  |
| Hep1615-R | AAAGGGCAGGGACGTAATC |  |  |  |  |
