# Supplementary material for: Canine vector-borne parasites in the Galapagos
Source: Parasit Vectors. 2024 Dec 18;17:515. doi: 10.1186/s13071-024-06592-z (PMC11656949; doi:10.1186/s13071-024-06592-z)
Supplement: Supplementary file 2 — Supplementary Material 2. BLAST comparisons between the obtained sequences and the GenBank sequences. [file 13071_2024_6592_MOESM2_ESM.docx]

BLAST comparisons between the obtained sequences and the GenBank sequences

| **Isolate** | **Organism** | **Gene** | **Sequence length (**bp**)** | **Query cover** | **E-value** | **Percent identity^*^ (%)** | **Similarity accession no** |
| --- | --- | --- | --- | --- | --- | --- | --- |
| D191 | *Dirofilaria immitis* | *cox1* | 689 | 99% | 2e-80 | 100 | MK250760 |
| D358 | *Dirofilaria immitis* | *cox1* | 678 | 99% | 4e-82 | 100 | LC107816 |
| D188 | *Dirofilaria immitis* | *cox1* | 659 | 99% | 0 | 100 | OQ359098 |
| D190 | *Dirofilaria immitis* | *cox1* | 659 | 99% | 0 | 100 | OQ359098 |
| D191 | *Dirofilaria immitis* | *cox1* | 659 | 99% | 0 | 100 | OQ359098 |
| D351 | *Dirofilaria immitis* | *cox1* | 648 | 98% | 0 | 100 | OP811228 |
| D358 | *Dirofilaria immitis* | *cox1* | 648 | 98% | 0 | 100 | OP811228 |
| D418 | *Dirofilaria immitis* | *cox1* | 648 | 98% | 0 | 100 | OP811228 |
| D632 | *Dirofilaria immitis* | *cox1* | 649 | 98% | 0 | 100 | OP494255 |
| D445 | *Dirofilaria immitis* | *cox1* | 671 | 69% | 0 | 83.93 | OQ726871 |
| D565 | *Dirofilaria immitis* | *cox1* | 678 | 99% | 0 | 100 | LC107816 |
| D604 | *Dirofilaria immitis* | *cox1* | 649 | 98% | 0 | 100 | OP494255 |
| ID081 | *Dirofilaria immitis* | *cox1* | 659 | 99% | 0 | 99.85 | OQ359098 |
| ID133 | *Dirofilaria immitis* | *cox1* | 477 | 77% | 0 | 100 | PP989676 |
| ID190 | *Dirofilaria immitis* | *cox1* | 671 | 69% | 0 | 100 | OQ726871 |
| D190 | *Dirofilaria immitis* | ITS2 | 209 | 40% | 4e-80 | 100 | OK632248 |
| D191 | *Dirofilaria immitis* | ITS2 | 222 | 44% | 1e-105 | 100 | MW590699 |
| D351 | *Dirofilaria immitis* | ITS2 | 223 | 43% | 2e-102 | 100 | OQ784647 |
| D418 | *Dirofilaria immitis* | ITS2 | 223 | 43% | 3e-102 | 99.53 | OQ784647 |
| D565 | *Dirofilaria immitis* | ITS2 | 223 | 46% | 1e-109 | 100 | OQ784647 |
| D632 | *Dirofilaria immitis* | ITS2 | 223 | 47% | 2e-107 | 100 | OQ784647 |
| ID081 | *Dirofilaria immitis* | ITS2 | 230 | 44% | 1e-105 | 100 | OQ784647 |
| ID133 | *Dirofilaria immitis* | ITS2 | 235 | 45% | 4e-110 | 99.56 | MZ677055 |
| ID190 | *Dirofilaria immitis* | ITS2 | 235 | 48% | 2e-112 | 100 | MZ677055 |
| ID199 | *Dirofilaria immitis* | ITS2 | 235 | 46% | 8e-92 | 100 | MN596214 |

^*^the percentage of the nucleotides that are the same between the two sequences
