# Supplementary material for: Canine vector-borne parasites in the Galapagos
Source: Parasit Vectors. 2024 Dec 18;17:515. doi: 10.1186/s13071-024-06592-z (PMC11656949; doi:10.1186/s13071-024-06592-z)
Supplement: Supplementary file 4 — Supplementary Material 4. GLM analysis results for Dirofilaria immitis and Babesia vogeli in dogs. [file 13071_2024_6592_MOESM4_ESM.docx]

GLM analysis results for *Dirofilaria immitis* and *Babesia vogeli* in dogs

|  | **Variable** | **Std. Error*** | **z value** | **Pr(>\|z\|)**** |
| --- | --- | --- | --- | --- |
| ***Dirofilaria immitis*** | | | | |
|  | Intercept | 5447.6 | -0.004 | 0.997 |
|  | Free | 0.4497 | -0.330 | 0.742 |
|  | Dogs from Isabela Island | 5447.6 | 0.003 | 0.997 |
|  | Dogs from San Cristóbal | 5447.6 | 0.003 | 0.997 |
|  | Dogs from Santa Cruz | 5600.9 | 0.000 | 1.000 |
|  | Housing | 0.62 | 0.540 | 0.589 |
|  | Sex | 0.41 | 0.968 | 0.333 |
|  |  |  |  |  |
| ***Babesia vogeli*** | | | | |
|  | Intercept | 738.34 | -0.023 | 0.92 |
|  | Free | 0.27 | 0.24 | 0.81 |
|  | Dogs from Isabela Island | 73834601 | 0.020 | 0.984 |
|  | Dogs from San Cristóbal | 73834598 | 0.020 | 0.984 |
|  | Dogs from Santa Cruz | 73.834.614 | 0.017 | 0.986 |
|  | Housing | 0.34029 | -0.170 | 0.865 |
|  | Sex | 0.24540 | -1093 | 0.275 |
|  |  |  |  |  |

*****Standard Error, ******Significant value (p=0.000)
