# Supplementary material for: Canine vector-borne parasites in the Galapagos
Source: Parasit Vectors. 2024 Dec 18;17:515. doi: 10.1186/s13071-024-06592-z (PMC11656949; doi:10.1186/s13071-024-06592-z)
Supplement: Supplementary file 5 — Supplementary Material 5. BLAST comparisons between the obtained sequences and the GenBank sequences. [file 13071_2024_6592_MOESM5_ESM.docx]

BLAST comparisons between the obtained sequences and the GenBank sequences

|  | **Isolate** | **Organism** | **Gene** | **Sequence length (bp)** | **Query cover (%)** | **E-value** | **Percent identity^*^ (%)** | **Similarity accession no** |
| --- | --- | --- | --- | --- | --- | --- | --- | --- |
| 1 | D004 | *Theileria orientalis* | 18S rDNA | 546 | 100 | 0 | 99.63 | CP056070 |
| 2 | D006 | *Theileria sergenti* | 18S rDNA | 541 | 100 | 0 | 100 | JQ723015 |
| 3 | D009 | *Babesia vogeli* | 18S rDNA | 764 | 96 | 0 | 99.78 | KT323932 |
| 4 | D010 | *Babesia vogeli* | 18S rDNA | 523 | 100 | 0 | 100 | KT333456 |
| 5 | D012 | *Theileria equi* | 18S rDNA | 560 | 99 | 0 | 99.82 | MG551915 |
| 6 | D131 | *Theileria equi* | 18S rDNA | 506 | 100 | 0 | 99.41 | MT093500 |
| 7 | D146 | *Theileria equi* | 18S rDNA | 560 | 99 | 0 | 100 | MG551915 |
| 8 | D147 | *Babesia vogeli* | 18S rDNA | 530 | 100 | 0 | 100 | KT333456 |
| 9 | D148 | *Babesia vogeli* | 18S rDNA | 527 | 100 | 0 | 99.43 | KT333456 |
| 10 | D149 | *Babesia vogeli* | 18S rDNA | 535 | 100 | 0 | 98.69 | KT333456 |
| 11 | D160 | *Theileria equi* | 18S rDNA | 557 | 100 | 0 | 100 | MT093500 |
| 12 | D162 | *Theileria equi* | 18S rDNA | 556 | 100 | 0 | 99.46 | MT093500 |
| 13 | D164 | *Theileria orientalis* | 18S rDNA | 550 | 99 | 0 | 99.45 | CP056070 |
| 14 | D170 | *Theileria orientalis* | 18S rDNA | 536 | 100 | 0 | 99.44 | CP056070 |
| 15 | D176 | *Theileria orientalis* | 18S rDNA | 480 | 100 | 0 | 99.38 | CP056070 |
| 16 | D177 | *Theileria orientalis* | 18S rDNA | 454 | 99 | 0 | 99.34 | CP056070 |
| 17 | D193 | *Hepatozoon canis* | 18S rDNA | 581 | 99 | 0 | 99.83 | MN393911 |
| 18 | D195 | *Babesia vogeli* | 18S rDNA | 483 | 93 | 0 | 99.56 | OM069367 |
| 19 | D201 | *Theileria orientalis* | 18S rDNA | 549 | 100 | 0 | 99.45 | MH208634 |
| 20 | D204 | *Theileria orientalis* | 18S rDNA | 562 | 99 | 0 | 99.64 | MH208641 |
| 21 | D207 | *Theileria orientalis* | 18S rDNA | 531 | 99 | 0 | 99.82 | CP056066 |
| 22 | D208 | *Theileria orientalis* | 18S rDNA | 463 | 100 | 0 | 99.78 | MH208641 |
| 23 | D224 | *Babesia vogeli* | 18S rDNA | 528 | 100 | 0 | 99.81 | OQ727065 |
| 24 | D249 | *Theileria equi* | 18S rDNA | 556 | 100 | 0 | 99.82 | MT093500 |
| 25 | D269 | *Theileria equi* | 18S rDNA | 531 | 100 | 0 | 100 | MT767169 |
| 26 | D282 | *Theileria* sp. | 18S rDNA | 560 | 99 | 0 | 99.46 | KJ020546 |
| 27 | D305 | *Babesia vogeli* | 18S rDNA | 530 | 97 | 0 | 99.61 | PP859319 |
| 28 | D320 | *Theileria equi* | 18S rDNA | 516 | 100 | 0 | 100 | MN857679 |
| 29 | D347 | *Theileria equi* | 18S rDNA | 554 | 100 | 0 | 99.46 | MT093500 |
| 30 | D351 | *Theileria equi* | 18S rDNA | 506 | 100 | 0 | 99.41 | MT093500 |
| 31 | D397 | *Theileria equi* | 18S rDNA | 552 | 100 | 0 | 100 | LC431546 |
| 32 | D413 | *Babesia canis* | 18S rDNA | 529 | 98 | 0 | 100 | OP412824 |
| 33 | D414 | *Babesia canis* | 18S rDNA | 482 | 100 | 0 | 99.78 | MK872807 |
| 34 | D415 | *Babesia canis* | 18S rDNA | 455 | 100 | 0 | 99.78 | MK872807 |
| 35 | D416 | *Theileria equi* | 18S rDNA | 529 | 100 | 0 | 99.62 | LC431546 |
| 36 | D449 | *Theileria equi* | 18S rDNA | 545 | 100 | 0 | 100 | LC431546 |
| 37 | D463 | *Theileria equi* | 18S rDNA | 583 | 100 | 0 | 99.76 | OQ692565 |
| 38 | D465 | *Theileria equi* | 18S rDNA | 355 | 100 | 0 | 100 | MZ327268 |
| 39 | D466 | *Theileria equi* | 18S rDNA | 810 | 100 | 0 | 100 | PQ44782 |
| 40 | D473 | *Theileria equi* | 18S rDNA | 454 | 100 | 0 | 100 | MN857679 |
| 41 | D483 | *Theileria orientalis* | 18S rDNA | 454 | 99 | 0 | 99.34 | CP056070 |
| 42 | D500 | *Theileria orientalis* | 18S rDNA | 463 | 100 | 0 | 99.78 | MH208641 |
| 43 | D510 | *Babesia vogeli* | 18S rDNA | 532 | 96 | 0 | 99.81 | MT674936 |
| 44 | D537 | *Theileria haneyi* | 18S rDNA | 460 | 96 | 0 | 99.34 | ON429011 |
| 45 | D544 | *Theileria haneyi* | 18S rDNA | 425 | 96 | 0 | 99.34 | ON429011 |
| 46 | D622 | *Theileria equi* | 18S rDNA | 916 | 98 | 1e-151 | 99.34 | PQ44781 |
| 47 | D625 | *Babesia vogeli* | 18S rDNA | 527 | 100 | 0 | 100 | KT333456 |
| 48 | ID015 | *Babesia vogeli* | 18S rDNA | 525 | 100 | 0 | 99.43 | KT333457 |
| 49 | ID020 | *Theileria equi* | 18S rDNA | 501 | 100 | 0 | 99.6 | MT093500 |
| 50 | ID023 | *Theileria equi* | 18S rDNA | 520 | 76 | 0 | 97.22 | MZ318530 |
| 51 | ID024 | *Theileria equi* | 18S rDNA | 297 | 99 | 1e-139 | 97.64 | MZ327062 |
| 52 | ID025 | *Theileria equi* | 18S rDNA | 454 | 100 | 0 | 100 | MN857679 |
| 53 | ID031 | *Babesia vogeli* | 18S rDNA | 532 | 96 | 0 | 99.81 | MT674936 |
| 54 | ID039 | *Babesia vogeli* | 18S rDNA | 533 | 100 | 0 | 100 | MN067709 |
| 55 | ID059 | *Babesia vogeli* | 18S rDNA | 522 | 100 | 0 | 100 | MN067709 |
| 56 | ID069 | *Theileria equi* | 18S rDNA | 486 | 100 | 0 | 99.79 | MN857679 |
| 57 | ID074 | *Babesia vogeli* | 18S rDNA | 533 | 100 | 0 | 99.81 | KT333456 |
| 58 | ID109 | *Babesia vogeli* | 18S rDNA | 532 | 100 | 0 | 99.87 | KT333456 |
| 59 | ID119 | *Babesia vogeli* | 18S rDNA | 530 | 100 | 0 | 99.43 | KT333456 |
| 60 | ID135 | *Babesia vogeli* | 18S rDNA | 533 | 99 | 0 | 99.62 | KT333456 |
| 61 | ID163 | *Babesia vogeli* | 18S rDNA | 531 | 100 | 0 | 99.44 | KT333456 |
| 62 | ID190 | *Hepatozoon canis* | 18S rDNA | 613 | 99 | 0 | 99.32 | MF588667 |
| 63 | ID191 | *Babesia vogeli* | 18S rDNA | 528 | 100 | 0 | 100 | MN067709 |
| 64 | SD017 | *Babesia vogeli* | 18S rDNA | 517 | 100 | 0 | 100 | KT333456 |
| 65 | SD119 | *Babesia vogeli* | 18S rDNA | 520 | 100 | 0 | 99.42 | KT333456 |
| 66 | SD209 | *Theileria orientalis* | 18S rDNA | 586 | 99 | 0 | 99.82 | MH208641 |
| 67 | SD238 | *Babesia vogeli* | 18S rDNA | 513 | 100 | 0 | 99.03 | KT333456 |
| 68 | D010 | *Babesia vogeli* | 18S rDNA | 530 | 100 | 0 | 100 | KT333456 |
| 69 | D147 | *Babesia vogeli* | 18S rDNA | 340 | 99 | 8e-172 | 99.7 | KT333456 |
| 70 | D195 | *Babesia vogeli* | 18S rDNA | 300 | 100 | 7e-142 | 100 | KY290979 |
| 71 | D208 | *Babesia vogeli* | 18S rDNA | 364 | 95 | 1e-150 | 95.66 | MH143391 |
| 72 | D224 | *Babesia vogeli* | 18S rDNA | 339 | 100 | 3e-146 | 100 | AY371194 |
| 73 | D305 | *Babesia canis* | 18S rDNA | 340 | 99 | 5e-174 | 100 | AY371194 |
| 74 | D415 | *Babesia vogeli* | 18S rDNA | 364 | 96 | 1e-170 | 99.41 | AY371195 |
| 75 | D622 | *Babesia canis* | 18S rDNA | 486 | 97 | 3e-176 | 100 | MN078323 |
| 76 | D625 | *Babesia canis* | 18S rDNA | 486 | 100 | 2e-177 | 99.43 | MN078323 |
| 77 | ID015 | *Babesia canis* | 18S rDNA | 486 | 100 | 8e-177 | 100 | MN078323 |
| 78 | ID020 | *Babesia vogeli* | 18S rDNA | 451 | 99 | 4e-165 | 98,25 | MT821127 |
| 79 | ID023 | *Babesia vogeli* | 18S rDNA | 451 | 99 | 1e-174 | 99.42 | MT821127 |
| 80 | ID024 | *Babesia canis* | 18S rDNA | 486 | 100 | 8e-177 | 100 | MN078323 |
| 81 | ID025 | *Babesia vogeli* | 18S rDNA | 451 | 98 | 0 | 97.01 | MT821127 |
| 82 | ID031 | *Babesia canis* | 18S rDNA | 534 | 98 | 2e-137 | 98.60 | KT272401 |
| 83 | ID039 | *Babesia vogeli* | 18S rDNA | 451 | 98 | 0 | 98.21 | MT821127 |
| 84 | ID059 | *Babesia vogeli* | 18S rDNA | 842 | 100 | 6e-138 | 98.27 | MG041384 |
| 85 | ID069 | *Babesia vogeli* | 18S rDNA | 451 | 98 | 0 | 97.01 | MT821127 |
| 86 | ID074 | *Babesia canis* | 18S rDNA | 486 | 99 | 3e-146 | 98.36 | MN078323 |
| 87 | ID109 | *Babesia canis* | 18S rDNA | 486 | 99 | 3e-146 | 98.36 | MN078323 |
| 88 | ID119 | *Babesia vogeli* | 18S rDNA | 451 | 99 | 0 | 97.19 | MT821127 |
| 89 | ID135 | *Babesia vogeli* | 18S rDNA | 451 | 98 | 2e-177 | 98.34 | MT821127 |
| 90 | ID163 | *Babesia vogeli* | 18S rDNA | 451 | 100 | 5e-164 | 96.39 | MT821127 |
| 91 | ID191 | *Babesia vogeli* | 18S rDNA | 451 | 99 | 5e-179 | 98.89 | MT821127 |
| 92 | SD017 | *Babesia canis* | 18S rDNA | 351 | 100 | 5e-174 | 99.42 | AY072926 |
| 93 | SD119 | *Babesia vogeli* | 18S rDNA | 451 | 98 | 9e-167 | 97.99 | MT821127 |
| 94 | SD209 | *Babesia canis* | 18S rDNA | 351 | 100 | 8e-177 | 99.71 | AY072926 |
| 95 | SD238 | *Babesia vogeli* | 18S rDNA | 342 | 98 | 2e-172 | 99.70 | MN067709 |
| 96 | D009 | *Babesia vogeli* | *cytb* | 503 | 100 | 0 | 99.60 | MK888706 |
| 97 | D010 | *Babesia vogeli* | *cytb* | 559 | 100 | 0 | 100 | MZ603879 |
| 98 | D146 | *Babesia vogeli* | *cytb* | 559 | 100 | 0 | 100 | MZ603879 |
| 99 | D147 | *Babesia vogeli* | *cytb* | 559 | 100 | 0 | 100 | MZ603879 |
| 100 | D148 | *Babesia vogeli* | *cytb* | 559 | 100 | 0 | 100 | MZ603879 |
| 101 | D149 | *Babesia vogeli* | *cytb* | 559 | 100 | 0 | 100 | MZ603879 |
| 102 | D170 | *Babesia vogeli* | *cytb* | 559 | 100 | 0 | 100 | MZ603879 |
| 103 | D195 | *Babesia vogeli* | *cytb* | 559 | 100 | 0 | 100 | MZ603879 |
| 104 | D224 | *Babesia vogeli* | *cytb* | 526 | 100 | 0 | 100 | MZ603878 |
| 105 | D282 | *Babesia vogeli* | *cytb* | 559 | 100 | 0 | 100 | MZ603879 |
| 106 | D305 | *Babesia vogeli* | *cytb* | 526 | 100 | 0 | 100 | MZ603878 |
| 107 | D415 | *Babesia vogeli* | *cytb* | 526 | 100 | 0 | 100 | MZ603878 |
| 108 | D416 | *Babesia vogeli* | *cytb* | 542 | 97 | 0 | 100 | MZ603876 |
| 109 | D510 | *Babesia vogeli* | *cytb* | 559 | 100 | 0 | 100 | MZ603879 |
| 110 | D625 | *Babesia vogeli* | *cytb* | 528 | 95 | 0 | 100 | MZ577087 |
| 111 | ID015 | *Babesia vogeli* | *cytb* | 559 | 100 | 0 | 100 | MZ603879 |
| 112 | ID031 | *Babesia vogeli* | *cytb* | 559 | 100 | 0 | 100 | MZ603879 |
| 113 | ID059 | *Babesia vogeli* | *cytb* | 559 | 100 | 0 | 100 | MZ603879 |
| 114 | ID074 | *Babesia vogeli* | *cytb* | 559 | 100 | 0 | 100 | MZ603879 |
| 115 | ID109 | *Babesia vogeli* | *cytb* | 559 | 100 | 0 | 100 | MZ603879 |
| 116 | ID119 | *Babesia vogeli* | *cytb* | 559 | 100 | 0 | 100 | MZ603879 |
| 117 | ID135 | *Babesia vogeli* | *cytb* | 559 | 100 | 0 | 100 | MZ603879 |
| 118 | ID163 | *Babesia vogeli* | *cytb* | 559 | 100 | 0 | 100 | MZ603879 |
| 119 | ID190 | *Babesia vogeli* | *cytb* | 559 | 100 | 0 | 100 | MZ603879 |
| 120 | ID191 | *Babesia vogeli* | *cytb* | 559 | 100 | 0 | 100 | MZ603879 |
| 121 | SD017 | *Babesia vogeli* | *cytb* | 559 | 100 | 0 | 100 | MZ603879 |
| 122 | SD119 | *Babesia vogeli* | *cytb* | 555 | 100 | 0 | 99.76 | MZ603880 |
| 123 | SD209 | *Babesia vogeli* | *cytb* | 555 | 100 | 0 | 100 | MZ603880 |
| 124 | D009 | *Babesia vogeli* | *cox1* | 863 | 100 | 0 | 100 | MZ577091 |
| 125 | D010 | *Babesia vogeli* | *cox1* | 863 | 100 | 0 | 100 | MZ577091 |
| 126 | D147 | *Babesia vogeli* | *cox1* | 863 | 100 | 0 | 100 | MZ577091 |
| 127 | D148 | *Babesia vogeli* | *cox1* | 863 | 100 | 0 | 100 | MZ577091 |
| 128 | D149 | *Babesia vogeli* | *cox1* | 863 | 100 | 0 | 100 | MZ577091 |
| 129 | D195 | *Babesia vogeli* | *cox1* | 863 | 100 | 0 | 100 | MZ577091 |
| 130 | D224 | *Babesia vogeli* | *cox1* | 852 | 100 | 0 | 99.77 | KC207825 |
| 131 | D282 | *Babesia vogeli* | *cox1* | 548 | 100 | 0 | 100 | MZ577086 |
| 132 | D305 | *Babesia vogeli* | *cox1* | 863 | 100 | 0 | 100 | MZ577091 |
| 133 | D415 | *Babesia vogeli* | *cox1* | 554 | 100 | 0 | 100 | MK888706 |
| 134 | D416 | *Babesia vogeli* | *cox1* | 863 | 100 | 0 | 100 | MZ577091 |
| 135 | D510 | *Babesia vogeli* | *cox1* | 863 | 100 | 0 | 100 | MZ577091 |
| 136 | ID015 | *Babesia vogeli* | *cox1* | 863 | 100 | 0 | 100 | MZ577092 |
| 137 | ID031 | *Babesia vogeli* | *cox1* | 863 | 100 | 0 | 100 | MZ577093 |
| 138 | ID059 | *Babesia vogeli* | *cox1* | 554 | 100 | 0 | 100 | MK888706 |
| 139 | ID074 | *Babesia vogeli* | *cox1* | 863 | 100 | 0 | 100 | MZ577091 |
| 140 | ID109 | *Babesia vogeli* | *cox1* | 863 | 100 | 0 | 100 | MZ577091 |
| 141 | ID119 | *Babesia vogeli* | *cox1* | 863 | 100 | 0 | 100 | MZ577091 |
| 142 | ID135 | *Babesia vogeli* | *cox1* | 863 | 100 | 0 | 100 | MZ577091 |
| 143 | ID163 | *Babesia vogeli* | *cox1* | 863 | 100 | 0 | 100 | MZ577091 |
| 144 | ID190 | *Babesia vogeli* | *cox1* | 554 | 100 | 0 | 100 | MK888706 |
| 145 | ID191 | *Babesia vogeli* | *cox1* | 548 | 100 | 0 | 100 | MZ577086 |
| 146 | SD017 | *Babesia vogeli* | *cox1* | 548 | 100 | 0 | 100 | MZ577086 |
| 147 | SD209 | *Babesia vogeli* | *cox1* | 554 | 100 | 0 | 100 | MK888706 |

^*^the percentage of the nucleotides that are the same between the two sequences
